# A trade‐off: Antimicrobial resistance and COVID‐19

**DOI:** 10.1111/bioe.12928

**Published:** 2021-08-31

**Authors:** Tess Johnson

**Affiliations:** ^1^ Oxford Uehiro Centre for Practical Ethics University of Oxford Oxford UK

**Keywords:** antimicrobial resistance, COVID‐19, distributive justice, pandemic ethics, trade‐off

## Abstract

As we combat the COVID‐19 pandemic, both the prescription of antimicrobials and the use of biocidal agents have increased in many countries. Although these measures can be expected to benefit existing people by, to some extent, mitigating the pandemic's effects, they may threaten long‐term well‐being of existing and future people, where they contribute to the problem of antimicrobial resistance (AMR). A trade‐off dilemma thus presents itself: combat COVID‐19 using these measures, or stop using them in order to protect against AMR. Currently, I argue, we are choosing to continue with these measures, and thus to prioritize combatting COVID‐19, without adequate ethical reflection on the AMR‐associated costs of these measures. I discuss the magnitude of the possible costs and benefits involved in making the trade‐off in favour of COVID‐19, and their distribution. I highlight two salient aspects of distribution that can help determine whether combatting COVID‐19 whilst exacerbating AMR produces justly distributed costs and benefits: distribution between current and future populations, and distribution between existing geographical populations. Adopting this account, I argue that based on the magnitude and distribution of costs and benefits of combatting COVID‐19, we have good reason to rethink this trade‐off, and instead consider prioritizing protecting current and future people against AMR, but jettisoning measures against COVID‐19 that also exacerbate AMR.

## 1 INTRODUCTION

During the COVID‐19 pandemic, certainly in 2020 at least, comparatively little attention was dedicated to how our response to the pandemic may impact our ability to pursue certain other public health goals. Certainly, there are some examples of work in the literature, especially more recently.^1^ However, the scope of work may be inadequate, considering the significance of these impacts where trade‐offs occur between, first, our current actions to combat COVID‐19 and protect existing people's current well‐being, and second, the maintenance of other public health goals that protect existing and future people's future health and well‐being. Antimicrobial resistance (AMR) is one threat to public health that may be significantly affected by our current actions. Whilst it is not the only important goal threatened by some measures to combat COVID‐19 (even within public health alone), it is a significant concern, given the urgency of antimicrobial stewardship. Trade‐offs such as this, among public health priorities, are also particularly salient for ethical analysis, as these are considered part of the responsibility of national health departments and other bodies, and as they concern commensurable values (e.g. health status), as opposed to trade‐offs that may be more difficult to compare (such as between environmental sustainability and health status).

AMR is the adaptation of disease‐causing microorganisms to treatment drugs such that these drugs are no longer effective at killing the pathogens. Resistant pathogens pose significant mortality risks to patients in healthcare settings, where they are vulnerable to co‐infection with the AMR pathogens. Some current actions to combat the spread of COVID‐19 may compromise efforts to protect against AMR, significantly affecting health and well‐being in the near future. Whilst not all measures against COVID‐19 face this issue, I focus in particular, on the examples of antimicrobial prescription for COVID‐19 co‐infections, and use of biocidal agents in household settings. The trade‐off must be assessed to ensure that our current course of action—that is, prioritizing combatting COVID‐19 by employing these measures—is the morally preferable course. Trade‐off dilemmas can be assessed using cost‐benefit analyses that account for the magnitude and distribution of harms and benefits (usually well‐being associated) if the trade‐off is made in favour of one option over the other. This cost‐benefit approach is central to a utilitarian framework, but also useful outside strict utilitarianism, where it is often employed in pluralist analyses of public health interventions. My focus here primarily on cost‐benefit analysis may be a limitation, given arguments against the adequacy of utilitarian analyses qua ethical analyses in some cases.^2^ However, on the whole, cost‐benefit analyses have proved a useful and accepted tool, particularly in public health ethics, in assessing the costs and benefits of a given intervention, at least as an informing aspect of ethical analysis.^3^ Up to now, a lack of ethical analysis of the trade‐off may have been acceptable, due to the immediacy of the COVID‐19 threat and our initial limited knowledge surrounding the dilemma. Now, however, our greater understanding of COVID‐19 and current behaviours that contribute to AMR mean that continued failure to undertake this analysis is negligent.

This paper introduces the COVID‐19‐AMR trade‐off firstly by identifying in Section 2 the collective responsibilities that we have both regarding contributions to the antimicrobial commons and pandemic control. Following this, in Section 3 I discuss how our current course of action prioritizes combatting COVID‐19, particularly when it comes to antimicrobial prescription and use of biocidal agents. In Section 3.1, I make the case for plausible assumptions regarding the magnitude of possible costs and benefits arising from prioritizing combatting COVID‐19 over antimicrobial protection. In Section 3.2, I discuss two aspects of the distribution of these costs and benefits: distribution between current versus future populations, and distribution among existing geographical populations. An analysis based on uncertain assumptions regarding the magnitudes of costs and benefits of prioritizing combatting COVID‐19 over AMR cannot be definitive; however, my argument, if correct, does offer a strong moral reason for changing our current AMR‐contributing behaviours in combatting COVID‐19 to better protect against AMR.

## 2 RESPONSIBILITY FOR THE ANTIMICROBIAL COMMONS

The World Health Organization (WHO) predicts that AMR will cause some 10 million deaths per year by 2050.^4^ Currently, WHO's antimicrobial stewardship framework recommends international programmes to promote the appropriate prescription and use of antimicrobials in medicine and agriculture.^5^ The goal is to preserve the antimicrobial commons, protecting against AMR. The commons is the global shared good of effective antimicrobial therapies, for which we have collective responsibility and to which we contribute via responsible use of antimicrobials and biocidal agents.^6^ Unlike *pure* public goods, the antimicrobial commons is not an entirely non‐competitive shared resource, because the more it is used, the more it is depleted and therefore unable to be used by others. This makes it a rivalrous, common pool resource, and international coordination to maintain it is more difficult: it is in each country's interest to continue supporting its own population via the use of antimicrobials in agriculture and medicine. Yet the more we use antimicrobials (especially ineffectively), the more selective pressure there is on microorganisms to evolve new mechanisms to resist the drugs, the effects of which are global. With innovation to develop new antimicrobial agents flagging, this resource is becoming increasingly competitive and non‐renewable.^7^

Attitudes of responsibility for the antimicrobial commons vary globally. For example, although one important aspect of antimicrobial stewardship is the responsible prescription of antimicrobials in medicine, adherence to guidelines is inconsistent. In South Africa, there is some evidence that healthcare inequalities and cultural resistance to ideas of collective responsibility for the global antimicrobial commons correlate with disproportionately high levels of antimicrobial prescription compared to many other countries.^8^ Prescription rates are particularly high in private healthcare, where national guidelines on the prescription of broad‐spectrum antibiotics do not apply. For lower‐income countries, however, prescribing behaviours resulting in higher antimicrobial use do not correlate with measures of the seriousness of AMR in that country according to the drug resistance index (DRI). Rather, a DRI indicating a large AMR problem in countries such as India is more affected by *lack of access* to effective antimicrobials to treat resistant infections in that country.^9^ In higher‐income countries, the correlation between the seriousness of AMR according to DRI measures and high antimicrobial prescription rates is stronger. In the UK for example, though the National Health Service (NHS) has pushed for comprehensive national guidelines regarding antimicrobial prescription, high levels of broad‐spectrum antibiotic prescription still occur (perhaps due to a continued emphasis on individual patient health and the reduced visibility of the health effects of AMR).^10^ This affects the DRI of the UK more than the spread of AMR infections due to lack of access to effective antimicrobials.

Some argue that international environment and trade law should hold countries accountable for their contributions to AMR.^11^ The Access Watch Reserve (AWaRe) database developed by the WHO, for example, if linked to the enforceable mandates expressed in its constitution, could set standards to protect and limit uses of newly‐developed antimicrobials for participating countries.^12^ Currently, AWaRe classifies and recommends antimicrobial drugs as first or second choice prescriptions according to their harms or benefits and current pathogen resistance to them. Purely advisory programmes such as this, however, have been shown inadequate to address clinicians’ prescription behaviours.^13^ Changing clinicians’ prescription behaviours to eliminate the *non‐beneficial* prescription of antimicrobials is one aspect of protecting the antimicrobial commons. The second aspect involves prescription behaviour when antimicrobials *can* be expected to provide clinical benefit to patients, but where this benefit—and patient expectations—must be weighed against costs to the antimicrobial commons. Clinicians’ decisions are complicated by their duty of care to their own patients, which they may consider to outweigh their responsibilities for the antimicrobial commons.^14^ In addition, clinicians’ perceptions of patients’ expectations to receive antimicrobials increase inappropriate prescription.^15^ Yet, where antimicrobial use can be expected to provide less individual benefit compared to the cost imposed on future people by depleting the antimicrobial commons, prescription may be morally impermissible. To avoid these difficulties, it has been suggested that instead of relying on clinicians’ decision‐making alone, we should consider taxing antimicrobials.^16^ Such a strategy aims to disincentivize beneficial but non‐essential uses of antimicrobials (i.e., use in cases where an infection is mild or self‐limiting) in high‐antimicrobial consumption, high‐income settings. It also provides revenue for funding antimicrobial research and development and conservation programmes.

One problem that continues to loom for the antimicrobial commons is the immediacy of other threats that temporarily increase antimicrobial use, at a cost to the commons. The COVID‐19 pandemic is one such threat.

## 3 AMR DURING THE COVID‐19 PANDEMIC

An international ‘grand bargain’ between countries to protect the antimicrobial commons does not yet exist, and is unlikely to in the near future, although work is being done to support such global agreements.^17^ Hospitals around the world are using different standards to prescribe antimicrobials, and to maintain general hospital hygiene using biocidal agents—particularly during the COVID‐19 pandemic. In the UK, the introduction of telehealth, prescription of antimicrobials to prevent or treat co‐infections, and increased use of biocidal agents in households and hospitals have already plausibly contributed to AMR.^18^

Ultimately, we are presented with a trade‐off between combatting COVID‐19 using antimicrobial measures, and refraining from doing so to better preserve the antimicrobial commons. Governments are responsible both for responding to pandemics (such as COVID‐19), and for protecting the public health of their current and future populations from other plausible threats (such as AMR). The trade‐off is currently being made in favour of prioritizing COVID‐19 protection, and with little ethical analysis.

### 3.1 Magnitude of possible costs and benefits of COVID‐19‐AMR trade‐off

First, let me consider the magnitude of possible costs and benefits associated with prioritizing combatting the COVID‐19 pandemic using current antimicrobial prescribing behaviours and biocide uses.

Even prior to COVID‐19, telemedicine and online healthcare appointments have been associated with increased and guideline‐non‐adherent antimicrobial prescribing practices.^19^ These prescriptions may either be non‐beneficial, or provide the patient with limited benefit, whilst imposing too great a cost to the antimicrobial commons in comparison. Particularly during a pandemic, where in‐person contact is additionally limited, clinicians may be unable to make adequately informed decisions regarding the trade‐off between a patient's current care and protection of the antimicrobial commons when remote appointments reduce their ability to assess the severity of a patient's illness. Prescription may benefit some patients who otherwise would have suffered from bacterial infections, ranging from less to more serious suffering, up to possible death. However, it is questionable how many of those individuals having telehealth appointments (as opposed to arriving at hospital with a serious bacterial infection) are in such need of antimicrobial prescriptions that they would significantly benefit from them, in comparison to the harm imposed by antimicrobial use on the AMR commons. In such cases, whilst individual benefit may be limited, the resulting over‐prescription of antimicrobials does pose significant and probable AMR‐associated costs, especially on a population‐wide scale. Cases of over‐prescription of antimicrobials in telehealth appointments have been documented in high‐income countries (HICs) including the UK and US, with a recent study showing that children in a US study were prescribed antibiotics for respiratory infections 52% of the time for telehealth appointments, 42% of the time for urgent care cases and 31% of the time for primary care visits. Among the telehealth antimicrobial prescriptions, only 59% of cases met prescribing guidelines.^20^ Additionally, although there was a significantly decreased number of medical appointments in the UK in April to August 2020 compared to the number in the previous year and a corresponding decrease in antibiotic prescription rates, the prescription rate was still 6.71% higher than expected.^21^ The costs of over‐prescription are serious: the reduction in effectiveness of broad‐spectrum antibiotics against diseases including tuberculosis, gonorrhoea, typhoid and Group B *Streptococcus* among others led to AMR‐associated costs of an estimated €9 billion in 2013 in Europe alone.^22^ These costs to healthcare systems—not to mention increasing mortality rates—are predicted to increase as more drug‐resistant strains emerge.

In hospital settings, increased prescribing rates have already been noted in the US during the COVID‐19 pandemic by the CDC.^23^ In fact, one recent metastudy showed that 72% of patients from hospital settings worldwide were prescribed antibiotics to treat suspected co‐infections in early 2020, whilst only 8% of patients actually had bacterial/fungal co‐infections.^24^ This discrepancy means that the benefit of antimicrobial prescriptions in such cases is itself minimal where co‐infection is not occurring. Such non‐essential prescribing decisions, even where they provide a small benefit to the individual, may contravene the current WHO guidance on AMR stewardship during the pandemic, where the expected benefit is not sufficiently great to outweigh the cost to the AMR commons.^25^ However, they also highlight potential feasibility issues with current guidance. The WHO's interim report recommends that patients with *moderate* COVID‐19 symptoms be left to self‐isolate or be admitted to hospitals on a case‐by‐case basis, and that antibiotics are not prescribed ‘unless there is clinical suspicion of a bacterial infection’.^26^ This guidance relies on the time, resources, and the ability to assess the severity of a patient's COVID‐19 and other symptoms, in order to determine the significance of the benefit they may receive via antimicrobial prescription, without in‐person consultation being possible, in some cases.

Whilst these prescription trends in UK and US telehealth and hospital settings may not be reflective of prescribing rates globally, or even in all HICs combatting the pandemic, insofar as these trends *are* illustrative of the issue, and insofar as these countries themselves have large enough populations to affect global AMR, they highlight an area of legitimate concern.

Biocides are cleaning agents that often use antimicrobials to decontaminate surfaces. Increased use in hospitals reduces the spread of existing resistant strains such as multidrug‐resistant *Staphylococcus aureus* (MRSA),^27^ which each year in the US kills more people than emphysema, HIV/AIDS, Parkinson's disease and homicide combined.^28^ Protection against resistant pathogens in hospital settings poses clear and significant benefit for vulnerable patients. However, biocide use is not limited to these settings in the current pandemic. Antimicrobials such as triclosan are often also present in household cleaning products, as well as everyday items from toys to toothpaste in the UK, Australia, Canada and other regions.^29^ Even where medically important antimicrobials are not included in biocides, other similar chemicals can indirectly contribute to AMR by causing ‘cross‐resistance’. This happens when resistance to one antimicrobial is caused by a pathogen's exposure to similar chemicals. Some household cleaning products are being used with greater frequency during the pandemic.^30^ This is also shown indirectly through scarcity in the supply of hand sanitizer products, as recorded in the UK setting.^31^ Household uses of some biocidal agents pose the discussed risks in terms of both expected contributions to AMR, and cross‐resistance.^32^ The benefit of using biocides is also reduced in household settings, assuming the households are representative of the broader population. The average individual is less vulnerable to being seriously affected by COVID‐19 (with one study in the first wave estimating a fatality rate of 0.14% for those under 60 years of age) compared to older individuals often in hospitals and other institutional care settings (with estimated fatality rates between 6.4% for those over 60 and 13.4% for those over 80).^33^ Thus, the benefits of biocide use are only probable and large in certain settings—primarily hospitals and institutional care settings. In contrast, the costs of such uses in addition to household uses are clear, with normal concentrations of triclosan reducing antimicrobial effectiveness by 100‐fold in a mouse model.^34^ Current increased uses of biocides, specifically in households, pose a significant cost and threat to the AMR commons without likely and significant benefit.

I turn now to discussing how the costs and benefits of the actions discussed here are distributed.

### 3.2 Distribution of costs and benefits of the COVID‐19‐AMR trade‐off

#### 3.2.1 Distribution across current versus future populations

Assessing the morally significant costs and benefits of our current actions can be difficult when these outcomes fall across time and generations. Often, short‐termism prevails in such assessments, either by default or due to political pressure. Short‐termism has been described as the maximization of short‐term performance at a cost to future competitiveness or viability (usually of a firm, as the term arises in business ethics).^35^ The concept can be usefully applied when we consider the current prioritization of combatting COVID‐19, which benefits current people, at a possible cost to their future selves and future generations.

Analyses have been proposed to assess outcome distribution over time and generations—particularly when it comes to future harms. Hilary Graham notes that it is ‘difficult for the public health community to direct attention to conditions for future health’ because ‘risk‐factor epidemiology pinpoints risks in temporal proximity to the individual […] and economic evaluations weigh policies according to their value to the current population’.^36^

I argue that we can and ought to adequately account for future harms without falling vulnerable to short‐termism, which risks turning the current population into a temporally privileged minority that unfairly benefits from temporal health inequalities.

Intergenerational justice demands that, in order to respect future persons, we treat harms to their well‐being as equally significant compared to harms to existing persons’ well‐being, at least once differences in certainty of those harms are accounted for by some discounting.^37^ That is, future individuals must be treated as the moral equals of existing individuals and be given ‘equal opportunities, and an equally high quality of life’.^38^

Moral equality of individuals over time also holds that we ought to treat harms that may occur to the same person, but in their future, as equally significant compared to current harms occurring to them. This contrasts with the ‘discounting’ that we commonly undertake, wherein ‘individuals discount risks in their own lives—future risks being regarded as of lower consequence than current risks’.^39^

Finally, environmental ethics uses the concept of sustainability to avoid discounting future harms, demanding the recognition of ‘responsibility to maintain a non‐declining set of opportunities based on possible [future] uses of the environment’.^40^ If such a responsibility exists, then the harm of reducing options for future people by our current actions certainly must be counted as a failure of justice. That is not to say that risking harm to future persons alone is determinative of the permissibility of our current actions. However, if both the magnitude and likelihood of harm are also expected to be greater, then these factors become more important. They also may combine with a failure of existing people to acknowledge responsibility for the future state of the world, and/or a failure to respect future persons as moral equals. Where these factors all indicate the burdening of future people with harms for which they may not experience corresponding benefits, they do make a strong case for the impermissibility of imposing the harm via our current actions.

Let us now apply this analysis to the COVID‐19‐AMR trade‐off. The uncertainty of the harms of prioritizing COVID‐19 and failing to protect the antimicrobial commons means there is some scope for legitimately applying a social discount rate. That is, we can reduce the economic or moral significance of effects on future people as they are increasingly more temporally distant, due to their reduced certainty—as long as the reduction is not hyperbolic, and as long as it does not discount future individuals’ *well‐being as such*.^41^ The future costs of AMR can be to a small extent discounted in this way, but given how we already see the effects of AMR and will do so in the near future, the discount rate should be low. In the COVID‐19 context, allowing continued over‐prescription of antibiotics for the supposed *prevention* of co‐infection, or in telehealth for beneficial but non‐essential treatment that imposes too great costs to the antimicrobial commons, is highly likely to contribute to these harms, based on our knowledge of the effects of antimicrobial use. The corresponding benefits of preventing the effects of co‐infection fall on current people, and not all those for whom antibiotics are prescribed as a preventative measure, at that (as some of these individuals would presumably not have been co‐infected anyway). As for the use of biocidal agents, the cross‐resistance effects detailed in the section above are highly likely to contribute to the predicted mortality rates from AMR in the future, posing a significant cost to future generations, whilst the benefits of reduced household spread of COVID‐19 fall primarily on the current population.^42^

Overall, we should consider all people—current and future, near and far away—as having equal moral status. In doing so, we assume basic moral equality as a grounding, above which an account of distributive justice may be applied. If this moral equality stands, then we have cause for concern when it comes to the COVID‐19‐AMR trade‐off. The harms caused by prioritizing COVID‐19 in the COVID‐19‐AMR trade‐off are significant, probable, and fall disproportionately on future people. This may contravene the requirements of a theory of justice grounded in basic moral equality of persons. For instance, sufficientarian and prioritarian accounts of distributive justice require that we not impose the costs of an act or intervention on the worst‐off, or at least not on those already below a minimum level of well‐being. Whilst the status of current versus future people in terms of well‐being levels can be difficult to determine, we may assume that, as a general, global trend, currently existing people who are worse off now are likely to remain so in the near future, whether this be a future with high levels of AMR or not. Similarly, those currently well off are likely to remain so. The pattern extends intergenerationally, wherein the descendants of these groups are likely to be similarly well off to their parents, following a trend of intergenerational transmission of poverty.^43^ I acknowledge that this is a limiting assumption, in that my following argument only stands if it is indeed the case that levels of well‐being can be expected to remain similar between generations, and thus that, due to the inapplicability of sufficientarian/prioritarian accounts of justice intergenerationally, we are to refer to the more basic idea of moral equality between individuals.

In contrast, the benefits of prioritizing COVID‐19 are quite certain, and fall primarily on existing people—although it must be acknowledged that there is some benefit to future people of combatting the current pandemic more effectively, given an anticipatable trickle‐down effect this has on them via the well‐being of current people. For instance, if the effects of the current pandemic are mitigated, healthcare systems world‐wide may be less depleted, leaving more healthcare resources for future generations. The same applies to economies, globally. The point remains that it is current people who benefit the most from our combatting COVID‐19 using the measures I am discussing. Their magnitude may be less than that of the future harms of AMR, depending on the effectiveness of antimicrobial prescription and use of biocides on increasing well‐being during the pandemic—a benefit that I have already discussed as limited in magnitude given the often non‐essential use of these measures.

Subscribers to Parfit's non‐identity problem^44^ may object to my analysis, claiming that we cannot truly say that even a future with depleted antimicrobial commons would be against the interests of future people, or harm those particular people. Mirroring Parfit's depletion scenario, some might argue that the counterfactual circumstances—that is, prioritizing AMR over combatting COVID‐19, and undertaking effective stewardship programmes that affect health behaviours—would cause different people (perhaps in number as well as identity) to exist in the future. If we assume that the lives of future people in a future with depleted antimicrobial commons would still be worth living, then it cannot be in those particular people's interests to not exist due to prioritizing effective AMR stewardship over COVID‐19 action. Indeed, on some accounts of harm, they would be harmed by not existing if we were to effectively undertake future‐changing AMR stewardship. However, as Parfit himself notes, there may be plausible claims regarding the impersonal costs of a (antimicrobial commons‐) depleted future, or impersonal benefits of a (antimicrobial commons‐) maintained future. That is, an antimicrobial commons‐depleted world may be worse off compared to a world (of different people) resulting from prioritizing AMR stewardship practices now.^45^

In that case, we can consider simply the impersonal costs and benefits involved in the COVID‐19‐AMR trade‐off. In aggregate, the impersonal harms associated with combatting COVID‐19 using measures that exacerbate AMR may be greater than the aggregate harms of doing the opposite.

If this is so, then it appears that an ethical analysis of the distribution of costs and benefits across current versus future populations using the concepts of intergenerational justice, moral equality and sustainability would yield a strong case against prioritizing combatting COVID‐19 in the COVID‐19‐AMR trade‐off.

#### 3.2.2 Distribution across existing geographical populations

The distribution of costs and benefits of making the COVID‐19‐AMR trade‐off in favour of combatting COVID‐19 (as we currently are) must also be assessed across existing populations. Geographical distribution of costs is especially notable for COVID‐19 and AMR as global phenomena whose effects defy borders. The global significance of both COVID‐19 and AMR seems to demand a theoretical approach of moral cosmopolitanism,^46^ requiring that the theory of justice subscribed to applies globally, rather than within national borders. Yet, we commonly undertake *spatial* social discounting as well as temporal discounting when it comes to the costs of current actions, resulting in an approach that employs a degree of moral nationalism.^47^ The well‐being costs of our actions for those who are far away are thus less salient than the effects for our neighbours. If we should avoid reliance on moral nationalism for issues with global morally relevant consequences, then the application of our account of justice should be applied globally. Two commonly accepted accounts in pandemic ethics that might be applied are prioritarianism, and sufficientarianism. Prioritarianism demands that the distribution of costs of an intervention not to fall on the worst‐off. Sufficientarianism demands that they not fall on those already below a minimum threshold of well‐being.^48^ Both theories are often applied in public health ethics, to ensure protection of the health of the global poor,^49^ based on an underlying commitment to basic moral equality.

Often the populations that are most geographically removed from those taking part in this academic discussion are the populations of low and middle‐income countries (LMICs). Many members of these populations are also the global worst‐off, and when applied simply, both sufficientarian and prioritarian accounts would demand that the burdens of our current actions at the very least do not primarily fall on those populations.

Based on emerging data, we can surmise that the prescription of antimicrobials during the COVID‐19 pandemic and increased use of biocidal agents has primarily occurred in well‐resourced HICs, where antimicrobials and biocidal agents are available.^50^ The health and well‐being benefits of these uses primarily accrue, then, to individuals who are already advantaged. This is because they have better‐resourced healthcare systems to support them, and often do not suffer from other untreated diseases that may exacerbate the effects of COVID‐19 infection. Additionally, given that elderly people are most vulnerable to serious health outcomes of COVID‐19, it is older populations (therefore, primarily those in HICs) who we can expect to be most benefitted by these measures. The same trend in benefit falling on the already‐advantaged may persist in access to antimicrobials and benefits of combatting COVID‐19 *within* HICs as well, with low‐SES background groups or individuals being less benefited than the better‐off. On the whole, however, the trend holds better globally. The vast majority of those who are already below a plausible minimum threshold of well‐being (say, the poverty line), are located in LMICs, and do not have as much access to the benefits of increased antimicrobial prescription and biocide use as those in HICs.^51^ Thus, the well‐being benefits of combatting COVID‐19 may seem to fall primarily on the better‐off, *contra* the requirements of prioritarianism and sufficientarianism.

Yet the contributions that current antimicrobial prescription behaviours and biocide uses make to AMR have more global costs. Indeed, it tends to be disadvantaged populations that suffer the most from both existing and emergent drug‐resistant pathogens. Resistant sexually transmitted diseases may primarily be caught by those already suffering from sexual exploitation,^52^ and resistant pathogens are both more easily spread and less easily treated in under‐resourced LMICs. As Irina Okeke and Robert Edelman note, ‘[p]oor infection control practices [, …] warm and humid tropical climate [and] the low level of sanitation in many developing countries, particularly in urban slums, provides an efficient means for the dissemination of these strains throughout the community’.^53^ It may be that the more that HICs trade‐off AMR stewardship efforts in favour of combatting COVID‐19, specifically, using current antimicrobial prescription and biocide uses, the more they push health burdens onto these other disadvantaged populations in LMICs (and to some extent, onto the worse‐off in HICs). The imposition of costs primarily on the worst‐off also runs against the requirements of prioritarian and sufficientarian conceptions of distributive justice.

It may be objected that, in fact, the problem of distribution is more nuanced than this. Insufficient efforts by already‐advantaged populations to curb the spread of COVID‐19 using measures such as increased antibiotic prescription and biocides may also impose costs on disadvantaged, geographically distant populations. Whilst it takes longer for well‐resourced healthcare systems to be overwhelmed by a given number of severe COVID‐19 cases, healthcare systems in LMICs may be quickly overwhelmed by the same number. Similarly, whilst vulnerable elderly populations are predominant in advantaged HICs, populations that are vulnerable due to existing untreated diseases are predominant in disadvantaged LMICs. Thus, the benefits of combatting COVID‐19 may fall more equally than it first appears.

Disadvantaged individuals in LMICs often suffer from poor nutrition, insecure housing, and comorbidities that may also increase their risk of death from COVID‐19. However, the same applies to the threat of AMR‐associated mortality, if the trade‐off is made in favour of combatting COVID‐19. By prioritizing COVID‐19, we impose the (future) costs of a depleted antimicrobial commons on the populations of countries already under‐prepared for combatting existing drug‐resistant pathogens. By prioritizing AMR, we impose the (current) costs of the increased spread of COVID‐19 and corresponding increased harms due to under‐resourcing and comorbidities on the same populations. Neither of these options is acceptable to a sufficientarian or prioritarian account of distributive justice. In this case, it appears that judgement comes down to the magnitude of possible harms, more than their distribution across existing geographical populations.

Although either COVID‐19‐associated harms or AMR‐associated harms will be exacerbated by making the trade‐off in one direction or the other, the magnitude of harm arising from AMR can be expected to be more significant than that of COVID‐19. In 2020, COVID‐19 killed around 1.6 million people.^54^ However, the scope of the pandemic is limited, with effective vaccines already being distributed. Whilst more deaths are expected throughout 2021, and the indirect effects of COVID‐19 may continue into the future, its effect is still likely to be less than that of exacerbated AMR in the future. Consider that AMR killed around 700,000 people per year globally as of 2015.^55^ If the exacerbation of these magnitudes of harm is equal in either direction of the COVID‐19‐AMR trade‐off, then the greatest overall harm to disadvantaged populations in LMICs occurs if we continue to prioritize combatting COVID‐19 over AMR stewardship.

## 4 CONCLUSION

During the COVID‐19 pandemic, we have chosen to prioritize combatting one virus over efforts to preserve the antimicrobial commons via effective antimicrobial stewardship. Here, I have discussed the magnitudes and distributions of costs and benefits associated with making the COVID‐19‐AMR trade‐off in favour of combatting COVID‐19.

The magnitude of expected harm is much greater for AMR than COVID‐19, according to plausible estimates. In terms of likelihood, the costs of AMR in 30 years are less certain than the costs of COVID‐19 in 2020–2021. However, we can extrapolate reliably from past and current effects of AMR, such that the predicted costs of AMR are still highly probable and of large magnitude, given current trajectories.

The harms of AMR fall primarily on future populations and our future selves. This runs counter to the standards set by moral equality and intergenerational justice. The harms fall around equally on the populations of LMICs compared to HICs, and thus advantaged and disadvantaged populations. In contrast, the benefits of combatting COVID‐19 fall primarily on current populations and the populations of HICs (though these effects are somewhat mitigated by the global nature of the pandemic).

If the analysis I have proposed here is adopted, it seems clear that we have strong moral reason *not* to make the COVID‐19‐AMR trade‐off in favour of combatting COVID‐19. This case is only made more convincing by how small some of the benefits of current antimicrobial prescription behaviours and biocide uses are, particularly where over‐prescription occurs and where biocides are used in an average household, as opposed to an institutional care setting.

One aspect of analysis I have not considered here is whether, where the costs of responsible antimicrobial stewardship are low, we (those combatting COVID‐19 using antimicrobials) may have duties of ‘easy rescue’—that is, taking on a small sacrifice in order to protect or promote significant broader interests or protect from significant harm to others.^56^ It may be that such a duty should be enforced by governments in HICs, through some of the legislative measures discussed in the antimicrobial stewardship section of this paper.

Through combatting this pandemic, the damage to the antimicrobial commons may already be done, but my analysis applies also to future pandemics of similar scale. We must not fall prone to short‐termism or spatial discounting. Rather, we must be informed by ethical analysis, and act on that analysis effectively using legislative tools, in order to avoid acting impermissibly where our pandemic responses affect the antimicrobial commons.

## CONFLICT OF INTEREST

The author declares no conflict of interest.

## Footnotes

1 See, for example, Bavli, I., Sutton, B., & Galea, S. (2020). Harms of public health interventions against covid‐19 must not be ignored. *BMJ, 371*, m4074.

2 Hansson, S. O. (2013). *The ethics of risk: Ethical analysis in an uncertain world*. Palgrave Macmillan.

3 Marseille, E., & Kahn, J. G. (2019). Utilitarianism and the ethical foundations of cost‐effectiveness analysis in resource allocation for global health. *Philosophy, Ethics, and Humanities in Medicine, 14*, Art.5.

4 UN Interagency Coordination Group on AMR. (2019). *No time to wait: Securing the future from drug‐resistant infections*. World Health Organization. https://www.who.int/antimicrobial-resistance/interagency-coordination-group/final-report/en/

5 Ibid.

6 Krockow, E. M., & Tarrant, C. (2019). The international dimensions of antimicrobial resistance: Contextual factors shape distinct ethical challenges in South Africa, Sri Lanka and the United Kingdom, *Bioethics, 33*(7), 756–765.

7 Hoffman, S. J., Savulescu, J., Giubilini, A., Kirchhelle, C., Rogers van Katwyk, S., Weldon, I., Campus, B., Harrison, M., Maslen, H., & McLean, A. (2020). Governing the global antimicrobial commons: Introduction to special issue. *Health Care Analysis, 28(1)*. https://doi.org/10.1007/s10728-019-00388-4

8 Paruk, F., Richards, G., Scribante, J., Baghwanjee, S., Mer, M., & Perrie, H. (2012). Antibiotic prescription practices and their relationship to outcome in South Africa: Findings of the prevalence of infection in South African intensive care units (PISA) study. *South African Medical Journal, 102*(7), 613–616.

9 Klein, E. Y., Tseng, K. K., Pant, S., & Laxminarayan, R. (2019). Tracking global trends in the effectiveness of antibiotic therapy using the Drug Resistance Index. *BMJ Global Health, 4*(2), e001315.

10 Krockow & Tarrant, op. cit. note 6.

11 Hoffman et al., op. cit. note 7.

12 Rogers Van Katwyk, S., Weldon, I., Giubilini, A., Kirchhelle, C., Harrison, M., McLean, A., Savulescu, J., & Hoffman, S. J. (2020). Making use of existing international legal mechanisms to manage the global antimicrobial commons: Identifying legal hooks and institutional mandates. *Health Care Analysis, 28*(1). https://doi.org/10.1007/s10728-020-00393-y; World Health Organization. (2019). *The AWaRe classification of antibiotics database*. World Health Organization. https://adoptaware.org/

13 Davey, P., Marwick, C. A., Scott, C. L., Charani, E., McNeil, K., Brown, E., Gould, I. A., Ramsay, C. R., & Michie, S. (2017). Interventions to improve antibiotic prescribing practices for hospital inpatients. *The Cochrane Database of Systematic Reviews, 2*(2), CD003543. https://doi.org/10.1002/14651858.CD003543.pub4

14 Rid, A., Littmann, J., & Buyx, A. (2019). Evaluating the risks of public health programs: Rational antibiotic use and antimicrobial resistance. *Bioethics, 33*(7), 734–748.

15 Dao, B., Douglas, T., Giubilini, A., Savulescu, J., Selgelid, M., & Faber, N. (2019). Impartiality and infectious disease: Prioritizing individuals versus the collective in antibiotic prescription. *AJOB Empirical Bioethics, 10*(1), 63–69.

16 Giubilini, A. (2019). Antibiotic resistance as a tragedy of the commons: An ethical argument for a tax on antibiotic use in humans. *Bioethics, 33*(7), 776–784.

17 Hoffman et al., op. cit. note 7.

18 Getahun, H., Smith, I., Trivedi, K., Paulin, S., & Balkhy, H. H. (2020). Tackling antimicrobial resistance in the COVID‐19 pandemic. *Bulletin of the World Health Organization, 98*(7), 441–508.

19 Hersh, A. L., Stenehjem, E., & Daines, W. (2019). RE: Antibiotic prescribing during pediatric direct‐to‐consumer telemedicine visits. *Pediatrics, 144*(2), e20191786B.

20 Ray, K. N., Shi, Z., Gidengil, C., Poon, S. J., Uscher‐Pines, L., & Mehrotra, A. (2019). Antibiotic prescribing during pediatric direct‐to‐consumer telemedicine visits. *Pediatrics, 143*(5), e20182491.

21 Armitage, R., & Nellums, L. M. (2020). Antibiotic prescribing in general practice during COVID‐19. *The Lancet Infectious Diseases, 22(6)*, *e144*. https://doi.org/10.1016/S1473-3099(20)30917-8

22 Oxford, J., & Kozlov, R. (2013). Antibiotic resistance—A call to arms for primary healthcare providers. *International Journal of Clinical Practice, 67*(Suppl. 180), 1–3.

23 Hsu, J. (2020). How Covid‐19 is accelerating the threat of antimicrobial resistance. *BMJ, 369*, m1983.

24 Rawson, T. M., Moore, L., Zhu, N., Ranganathan, N., Skolimowska, K., Gilchrist, M., Satta, G., Cooke, G., & Holmes, A. (2020). Bacterial and fungal coinfection in individuals with coronavirus: A rapid review to support COVID‐19 antimicrobial prescribing. *Clinical Infectious Diseases, 71*(9), 2459–2468. https://doi.org/10.1093/cid/ciaa530

25 World Health Organization. (2020). *Clinical management of COVID‐19 interim guidance—May 2020*. World Health Organization. https://www.who.int/publications-detail/clinical-management-of-covid-19

26 Ibid: 5.

27 Mitsuboshi, S., & Tsugita, M. (2019). Impact of alcohol‐based hand sanitizers, antibiotic consumption, and other measures on detection rates of antibiotic‐resistant bacteria in rural Japanese hospitals. *Journal of Infection and Chemotherapy, 25*(3), 225–228.

28 Infectious Diseases Society of America. (2011). Combating antimicrobial resistance: Policy recommendations to save lives. *Clinical Infectious Diseases, 52*(Suppl. 5), 397–428.

29 Westfall, C., Flores‐Mireles, A. L., Robinson, J. I., Lynch, A., Hultgren, S., Henderson, J., & Levin, P. A. (2019). The widely used antimicrobial triclosan induces high levels of antibiotic tolerance in vitro and reduces antibiotic efficacy up to 100‐fold in vivo. *Antimicrobial Agents and Chemotherapy, 63*(5), e02312‐18.

30 Suchomel, M., Eggers, M., Maier, S., Kramer, A., Dancer, S. J., & Pittet, D. (2020). Evaluation of World Health Organization—Recommended hand hygiene formulations. *Emerging Infectious Diseases, 26*(9), 2064–2068.

31 Health and Safety Executive, United Kingdom. (2021). *Hand sanitiser products and surface disinfectants during the coronavirus pandemic—Use, manufacture and supply*. Health and Safety Executive. https://www.hse.gov.uk/coronavirus/hand-sanitiser/index.htm

32 Chojnacki, M., Dobrotka, C., Osborn, R., Johnson, W., Young, M., Meyer, B., Laskey, E., Wozniak, R. A., Dewhurst, S., & Dunman, P. M. (2021). Evaluating the antimicrobial properties of commercial hand sanitizers. *Sphere, 3*(2), e00062‐21.

33 Verity, R., Okell, L., Dorigatti, I., Winskill, P., Whittaker, C., Imai, N., Cuomo‐Dannenburg, G., Thompson, H., Walker, P. G. T., Fu, H., Dighe, A., Griffin, J. T., Baguelin, M., Bhatia, S., Boonyasiri, A., Cori, A., Cucunubá, Z., FitzJohn, R., Gaythorpe, K. … Ferguson, N. (2020). Estimates of the severity of coronavirus disease 2019: A model‐based analysis. *The Lancet Infectious Diseases, 20*(6), 669–667.

34 Westfall et al., op. cit. note 29.

35 Scarborough, J. (2010). The ethical implications of short‐termism: Leadership failure in the executive suite. *International Journal of Value‐Based Management, 8*(1), 25–37.

36 Graham, H. (2010). Where is the future in public health? *The Milbank Quarterly, 88*(2), 149–168, p. 149.

37 It should be noted that although in some cases the reduced certainty of future circumstances appropriately discounts their weight in ethical analysis, there are other future circumstances that grow more predictable the further in the future they are, and that therefore there is no blanket‐rule that implies future harms should be considered less weighty than present harms (Cowen & Parfit, 1992). Cowen, T., & Parfit, D. (1992). Against the social discount rate. In P. Laslett & J. Fishkin (Eds.), *Justice Across the Generations: Philosophy, politics, and society, sixth series* (pp. 144–161). Yale University Press.

38 Parfit, D. (1984). *Reasons and persons*. Clarendon Press.

39 Pearce, D., Groom, B., Hepburn, C., & Koundouri, P. (2003). Valuing the future. *World Economics, 4*(2), 121–141, p. 122.

40 Norton, B. G. (2000). Biodiversity and environmental values: In search of a universal earth ethic. *Biodiversity & Conservation, 9*(8), 1029–1044, p. 1043.

41 Broome, J. (1994). Discounting the future. *Philosophy & Public Affairs, 23*(2), 128–156.

42 Although, note that where COVID‐19‐caused deaths are prevented, benefits fall on future descendants of the COVID‐19‐survivors, as well.

43 Bird, K. (2013). The intergenerational transmission of poverty: An overview. In A. Shepherd & J. Brunt (Eds.), *Chronic poverty*(pp. 60–84). Palgrave Macmillan.

44 Parfit, op. cit. note 38, ch. 16.

45 Then again, we may consider COVID‐19 futures as well. The benefit of existence in a future world in which COVID‐19 had been less effectively combatted due to the prioritization of protecting the antimicrobial commons would still hold for the particular individuals in that future. However, given the magnitude of difference made by AMR action compared to COVID‐19 action, it is likely that the impersonal benefits of AMR action are greater than the impersonal benefits of COVID‐19 action, for possible future worlds.

46 Kleingeld, P., & Brown, E. (2019). Cosmopolitanism. In E. Zalta (Ed.), *The Stanford encyclopedia of philosophy* (Winter 2019 ed.). https://plato.stanford.edu/archives/win2019/entries/cosmopolitanism/

47 Pearce et al., op. cit. note 39.

48 Reid, L. (2020). Antimicrobial resistance and social inequalities in health: Considerations of justice. In E. Jamrozik & M. Selgelid (Eds.), *Ethics and drug resistance: Collective responsibility for global public health* (pp. 257–278). Springer International Publishing.

49 Persad, G. (2019). Justice and public health. In A. Mastroianni, J. Kahn, & N. Kass (Eds.), *The Oxford handbook of public health ethics*. Oxford University Press. https://doi.org/10.1093/oxfordhb/9780190245191.013.4

50 Abelenda‐Alonso, G., Padulles, A., Rombauts, A., Gudiol, C., Pujol, M., Alvarea‐Pouso, C., Jodar, R., & Carratala, J. (2020). Antibiotic prescription during the COVID‐19 pandemic: A biphasic pattern. *Infection Control and Hospital Epidemiology, 41*(11), 1371–1372; Strathdee, S. A., Davies, S. C., & Marcelin, J. R. (2020). Confronting antimicrobial resistance beyond the COVID‐19 pandemic and the 2020 US Election. *The Lancet, 396*, 1050–1053.

51 A mitigating factor regarding benefits is the fact that overall reduced *global spread* of COVID‐19 does significantly benefit the populations of LMICs, which may be affected by measures in HICs.

52 Reid, op. cit. note 48, p. 263.

53 Okeke, I., & Edelman, R. (2001). Dissemination of antibiotic‐resistant bacteria across geographic borders. *Clinical Infectious Diseases, 33*(3), 364–369, p. 365.

54
*Coronavirus (COVID‐19) deaths*. (2020). Our World in Data. Retrieved December 9, 2020, from https://ourworldindata.org/covid-deaths

55 World Health Organization. (2020). *Health systems: Stewardship*. Retrieved August 28, 2020, from https://www.who.int/healthsystems/stewardship/en/

56 Giubilini, A., & Savulescu, J. (2020). Moral responsibility and the justification of policies to preserve antimicrobial effectiveness. In E. Jamrozik & M. Selgelid (Eds.), *Ethics and drug resistance: Collective responsibility for global public health* (pp. 141–154). Springer International Publishing.

